# Supplementary material for: The Association between Early-Onset Diagnosis and Clinical Outcomes in Triple-Negative Breast Cancer: A Systematic Review and Meta-Analysis
Source: Cancers (Basel). 2023 Mar 23;15(7):1923. doi: 10.3390/cancers15071923 (PMC10093252; doi:10.3390/cancers15071923)
Supplement: Supplementary file 1 [file cancers-15-01923-s001.zip › cancers-2296656-supplementary.pdf]

SUPPLEMENTAL FILE

Table S1: Search strategy concepts and terms used in Medline and EMBASE.

Table S2a-e: Meta-regression by later-onset age definition and confounding adjustment (based on Q5 score of Newcastle Ottawa Scale scale) for meta-analyses of (a) disease-free survival, (b) locoregional recurrence-free survival, (c) distant recurrence-free survival, (d) breast cancer-specific survival, and (e) pathological complete response.

Figure S1a-e: Funnel plot to assess publication bias for meta-analysis of (a) disease-free survival, (b) locoregional recurrence-free survival, (c) distant recurrence-free survival, (d) breast cancer-specific survival, and (e) pathological complete response.

Table S1: The concepts and terms used in Medline and EMBASE for search strategy

**Concept**	**Search terms**
Breast cancer	exp breast cancer/ OR exp breast tumor/ OR exp breast carcinoma/ OR exp breast carcinoma in situ/ OR (“breast cancer” or “breast neoplasm*” or “breast carcinoma” or “breast tumour*” or “breast tumor*” or “breast malignan*”).ab,ti.
Triple negative subtype	(“ER-/PR-/HER2-” or “ER/PR/HER2 negative” or “HR-/HER2-” or “HR/HER2 negative” or “receptor-negative” or “receptor negative” or “triple-negative” or “triple negative” or “triple-negative breast cancer” or “triple negative breast cancer” or “TNBC” or “triple-negative subtype” or “triple negative subtype” or “basal-like” or “basal like” or “basal-like breast cancer” or “basal like breast cancer” or “basal like subtype” or “basal-like subtype” or “BLBC” or “TN” or “TN subtype” or “ER-/HER2-” or “ER/HER2 negative” or “ER, PR, and HER2 status” or “ER, PR, HER2 negative”).ab,ti.
Clinical outcomes	exp cancer prognosis/ OR exp prognosis/ OR survival analysis/ OR exp progression free survival/ OR exp survival/ OR exp survival rate/ OR exp overall survival/ OR exp survivor/ OR exp recurrent disease/ OR exp cancer recurrence/ OR exp death/ OR exp mortality/ OR (“prognosis” or “survival” or “survivor*” or “overall survival” or “recur*” or “cancer recur*” or “mortality” or “death” or “all-cause mortality” or “outcome*” or “clinical outcome*” or “prognos*” “breast cancer specific survival” or “BCSS” “breast cancer survival” or “OS” or “RFS” or “DFS” “PFS” “recurrence” or “progression” “disease-free” “disease free”).ab,ti.
Young age (<40 years)	("young age at diagnosis" or "young women" or "very young women" or "young age" or "early age" or "young onset" or "early onset" or "young adult" or "early adult" or "young-onset" or "early-onset" or "under 40" or "under age 40" or "under the age of 40" or "under 40 years of age" or "under 40 years old" or "under 40 years" or "younger than 40" or "40 and younger" or "40 or younger"

	or "40 years and younger" or "40 years or younger" or "age 40 and younger" or "age 40 or younger" or "39 years and younger" or "39 years or younger" or "under 35" or "under age 35" or "under the age of 35" or "under 35 years of age" or "under 35 years old" or "under 35 years" or "younger than 35" or "35 and younger" or "35 or younger" or "35 years and younger" or "35 years or younger" or "under 30" or "under age 30" or "under the age of 30" or "under 30 years of age" or "under 30 years old" or "under 30 years" or "younger than 30" or "30 and younger" or "30 or younger" or "30 years and younger" or "30 years or younger" or "<40" or "≤40" or "<40 years" or "≤40 years" or "<40 years of age" or "≤40 years of age" or "<40 years old" or "≤40 years old" or "<35" or "≤35" or "<35 years" or "≤35 years" or "<35 years of age" or "≤35 years of age" or "<35 years old" or "≤35 years old" or "<30" or "≤30" or "<30 years" or "≤30 years" or "<30 years of age" or "≤30 years of age" or "<30 years old" or "≤30 years old").ab,ti.

Table S2a: Meta-regression by later-onset age group and confounding adjustment score for disease-free survival meta-analysis

**Variable**	**Coefficient**	**Standard Error**	**Lower 95% CI limit**	**Upper 95% CI limit**	**P-value**
**Later-onset age group (>60 years vs. ≥40 years)**	-.005685	.3336535	-.7096325	.6982624	0.987
**Confounding adjustment score (2 vs. 0 or 1)**	-.2192358	.1872002	-.6141937	.1757221	0.258

Table S2b: Meta-regression by later-onset age group and confounding adjustment score for locoregional recurrence-free survival meta-analysis

Variable	Coefficient	Standard Error	Lower 95% CI limit	Upper 95% CI limit	P-value
**Later-onset age group (>60 years vs. ≥40 years)**	.4204271	.3514168	-.4394587	1.280313	0.277
**Confounding adjustment score (2 vs. 0 or 1)**	-.4116476	.2181382	-.9454125	.1221172	0.108

Table S2c: Meta-regression by later-onset age group and confounding adjustment score for distant recurrence-free survival meta-analysis

Variable	Coefficient	Standard Error	Lower 95% CI limit	Upper 95% CI limit	P-value
**Later-onset age group (>60 years vs. ≥40 years)**	-.1552314	.306883	-.8808944	.5704315	0.629
**Confounding adjustment score (2 vs. 0 or 1)**	-.1274004	.2939823	-.8225581	.5677572	0.678

Table S2d: Meta-regression by later-onset age group and confounding adjustment score for breast cancer specific-survival survival meta-analysis

Variable	Coefficient	Standard Error	Lower 95% CI limit	Upper 95% CI limit	P-value
**Later-onset age group (>60 years vs. ≥40 years)**	-.488246	.1187751	-.7496682	-.2268237	**0.002**
**Confounding adjustment score (2 vs. 0 or 1)**	-.0631303	.1863762	-.3436755	.2174148	0.630

Table S2e: Meta-regression by later-onset age group and confounding adjustment score for overall survival meta-analysis

Variable	Coefficient	Standard Error	Lower 95% CI limit	Upper 95% CI limit	P-value
**Later-onset age group (>60 years vs. ≥40 years)**	-.5798395	.2293769	-1.071804	-.0878749	**0.024**
**Confounding adjustment score (2 vs. 0 or 1)**	.1285279	.2018576	-.3044136	.5614695	0.535

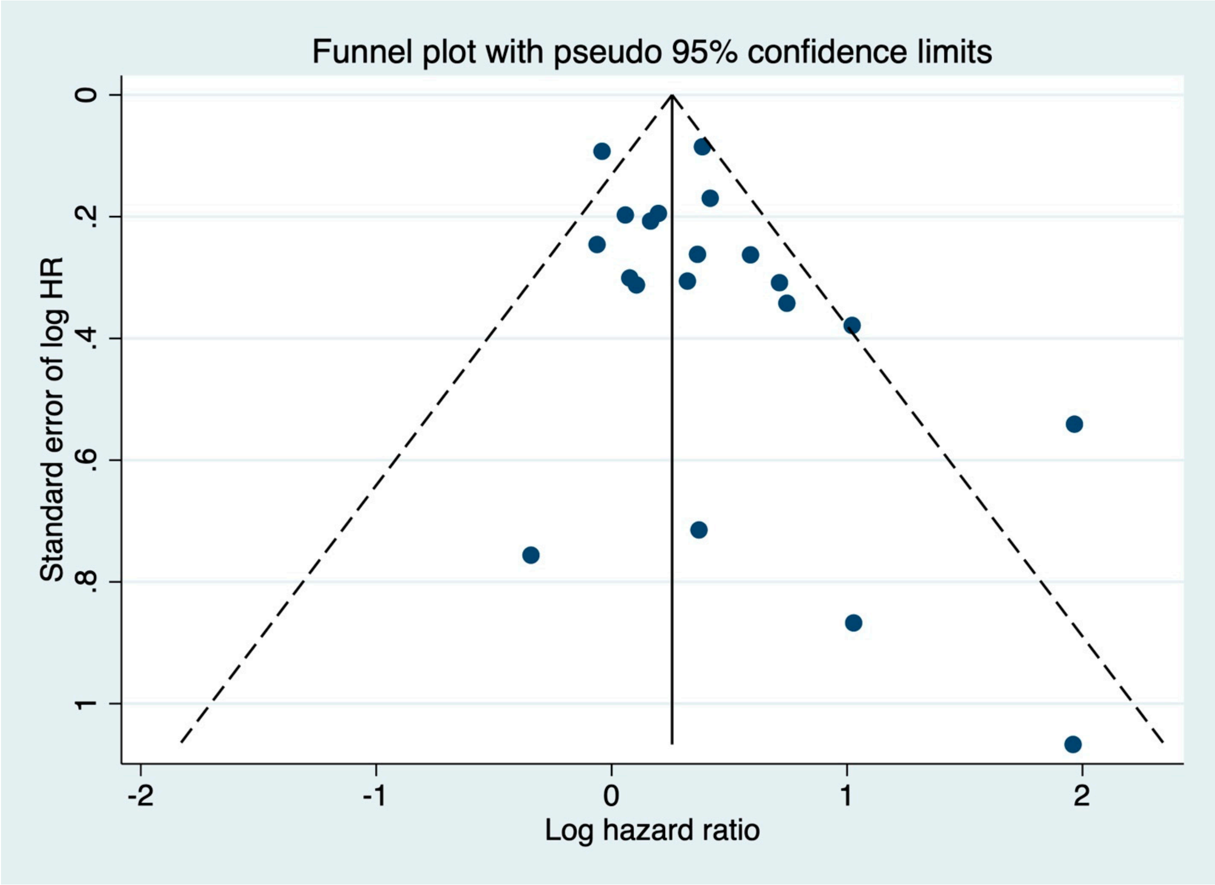

Figure S1a: Funnel plot to assess publications for meta-analysis of disease-free survival

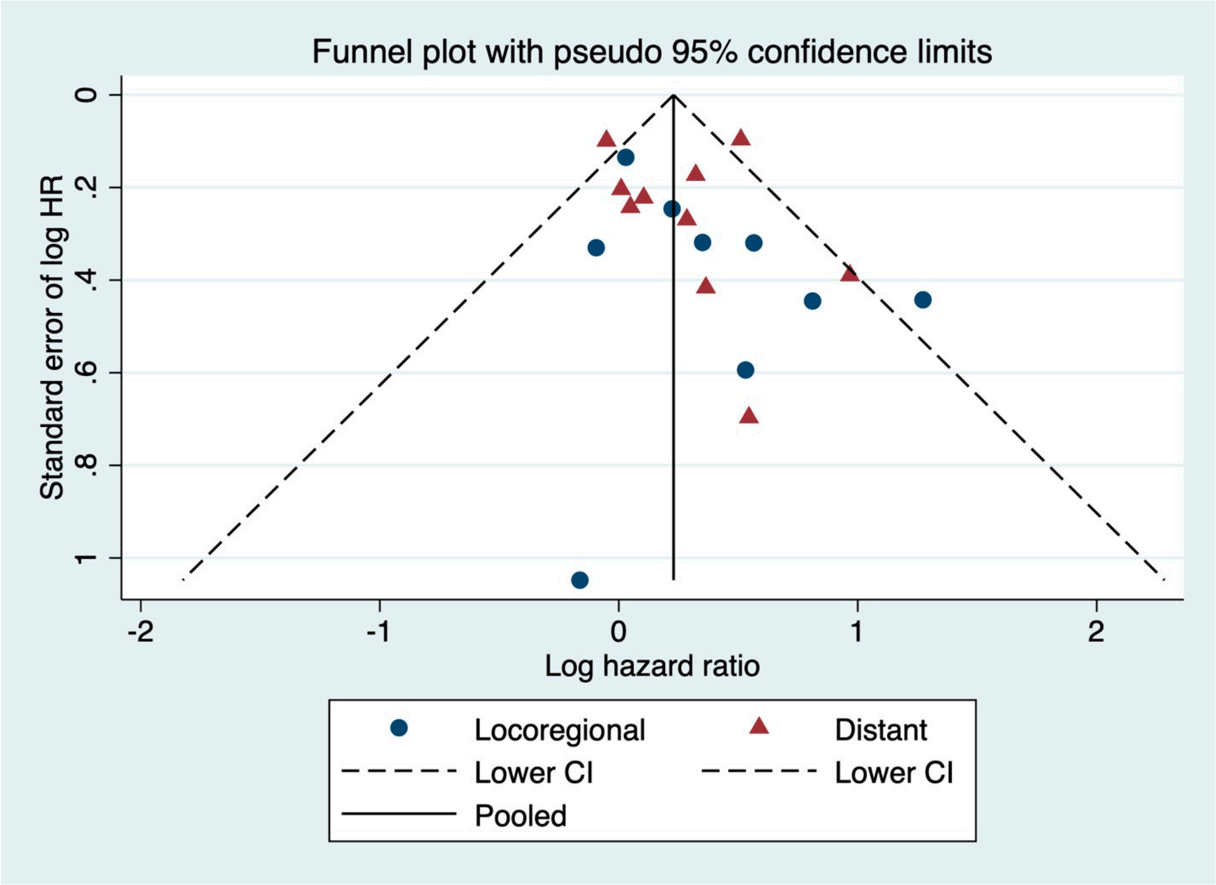

Figure S1b: Funnel plot to assess publications for meta-analysis of locoregional and distant recurrence-free survival

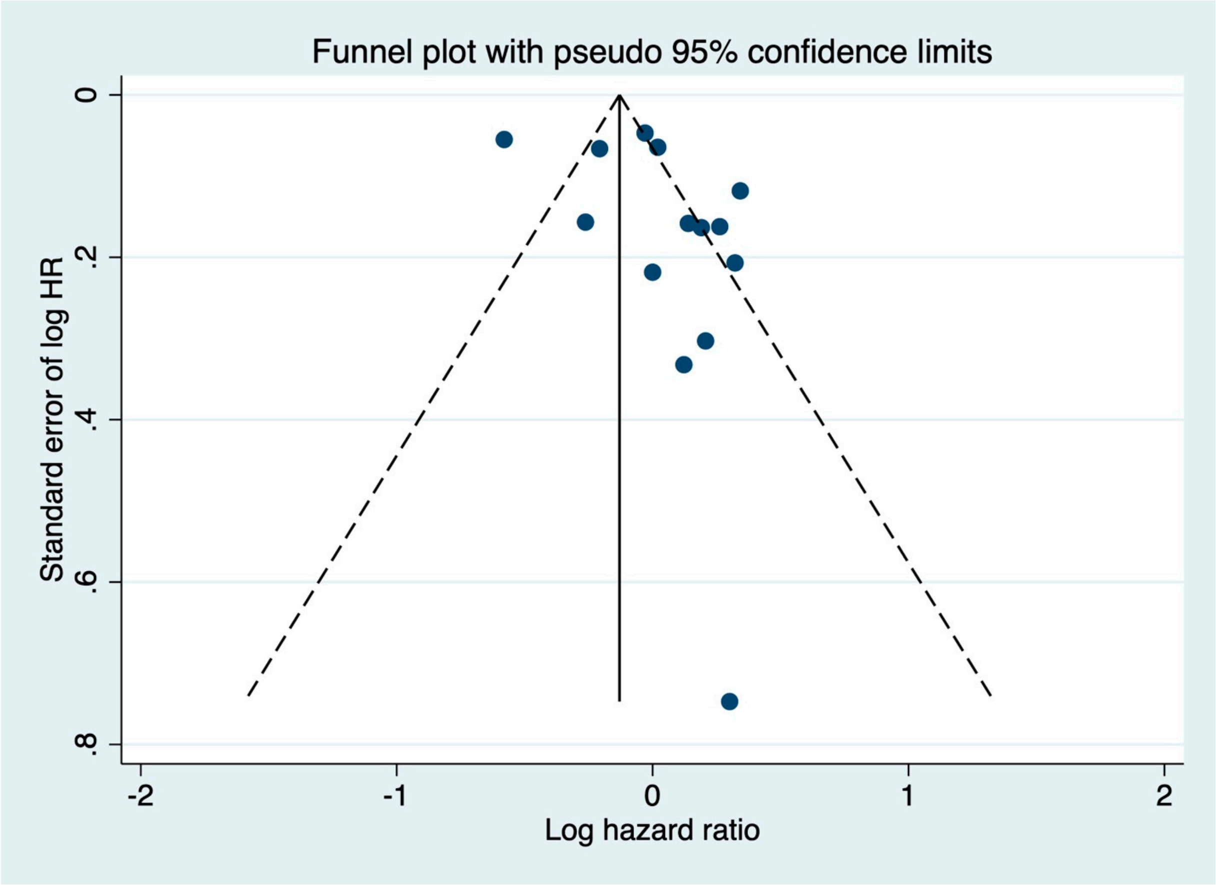

Figure S1c: Funnel plot to assess publications for meta-analysis of breast cancer specific survival

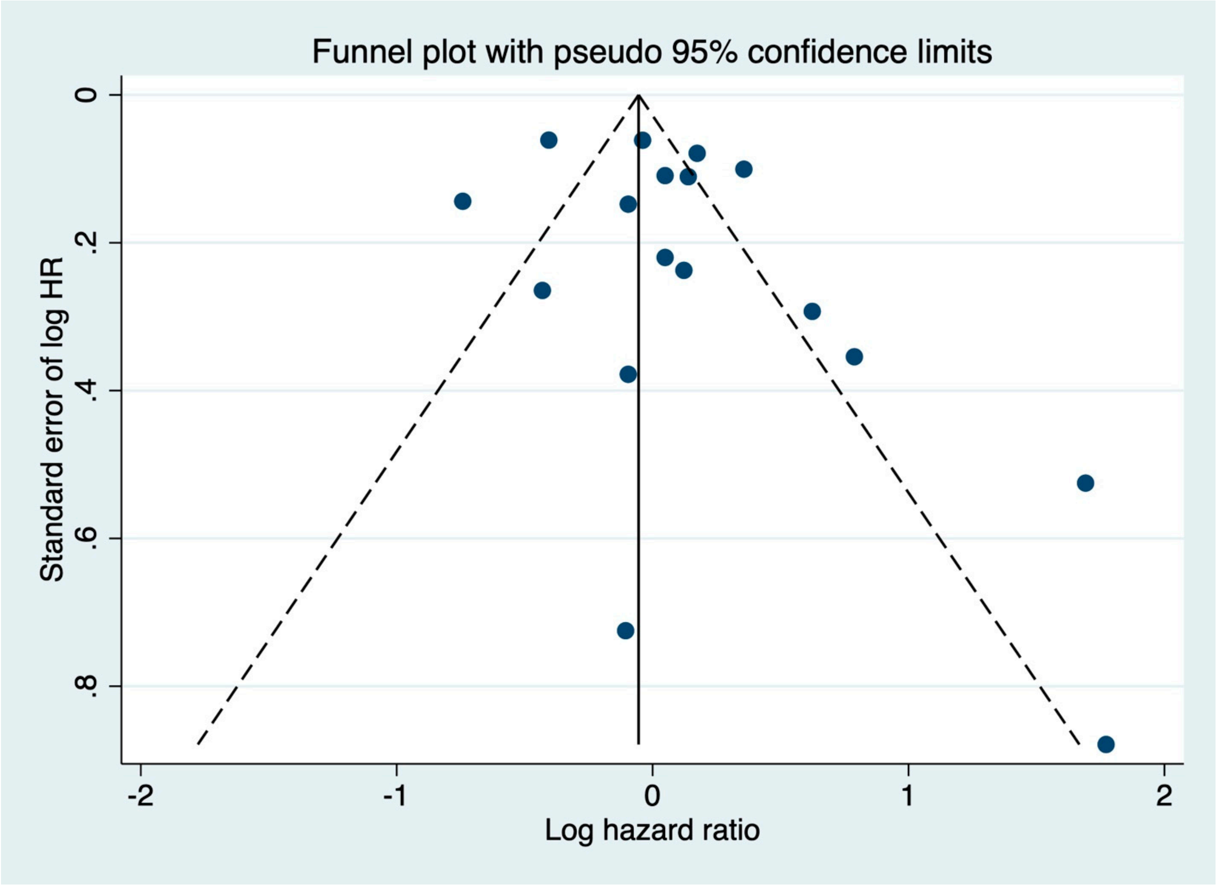

Figure S1d: Funnel plot to assess publications for meta-analysis of overall survival

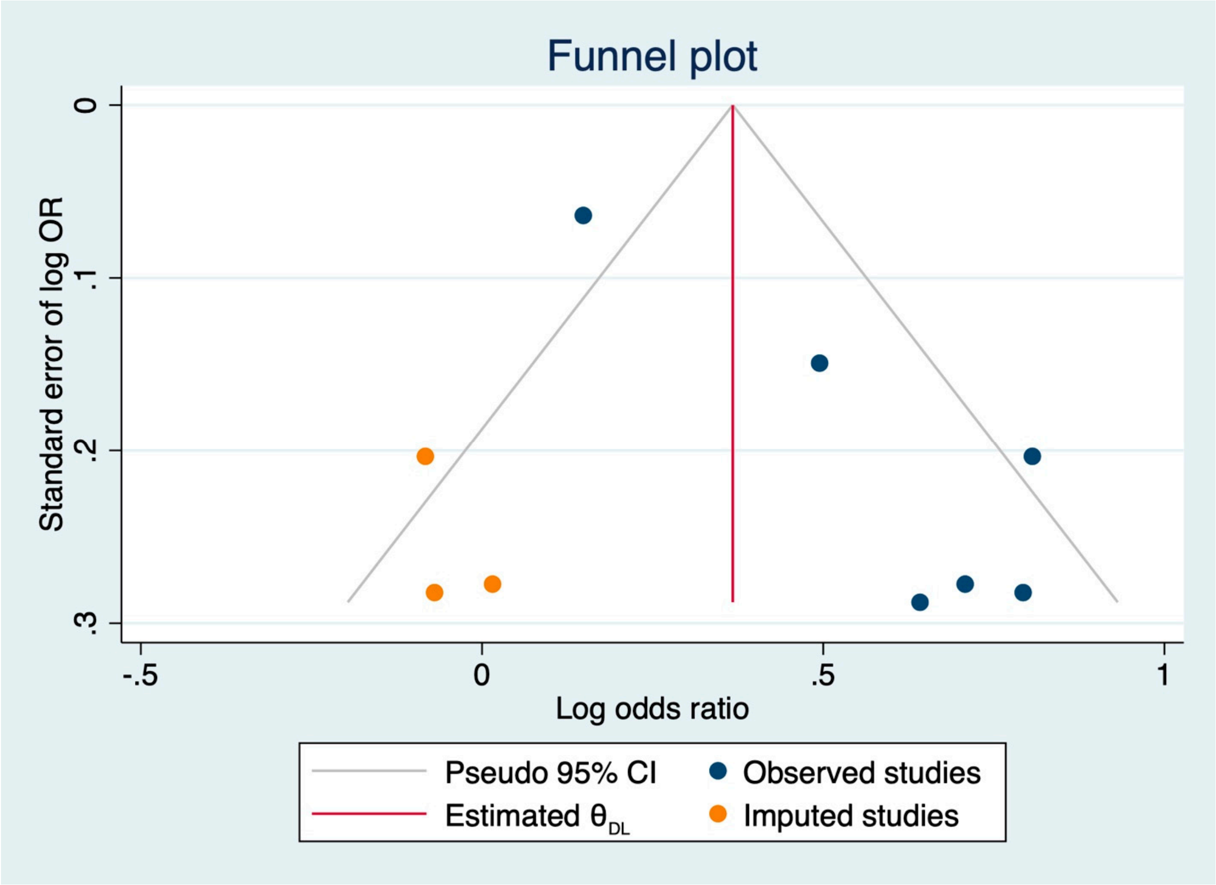

Figure S1e: Funnel plot to assess publications for meta-analysis of pathological complete response with inputted studies from trim and fill approach
